# Municipality‐Based Lifestyle Intervention of Childhood Overweight and Obesity: 4‐Years Follow‐Up on BMI Trajectories

**DOI:** 10.1111/cob.70082

**Published:** 2026-05-05

**Authors:** Mette Fogh, Jane Nautrup Østergaard, Helene Kirkegaard, Eeva‐Liisa Røssell, Helle Terkildsen Maindal, Gunnar Toft, Henrik Støvring, Jens Meldgaard Bruun

**Affiliations:** ^1^ Steno Diabetes Center Aarhus Aarhus University Hospital Aarhus Denmark; ^2^ Department of Clinical Medicine Aarhus University Aarhus Denmark; ^3^ National Institute of Public Health University of Southern Denmark Copenhagen Denmark; ^4^ Department of Public Health Aarhus University Aarhus Denmark; ^5^ Department of Biomedicine Aarhus University Aarhus Denmark; ^6^ Clinical Pharmacology, Pharmacy and Environmental Medicine University of Southern Denmark Odense Denmark; ^7^ Danish National Center for Obesity Aarhus Denmark

**Keywords:** childhood, health promotion, municipality‐based, obesity treatment

## Abstract

To examine 4‐year changes in Body Mass Index z‐score (BMIz) among children and adolescents with overweight or obesity who participated in municipality‐based interventions, and to assess whether effects varied by age, sex or baseline weight category. This matched cohort study included children aged 5–15 years. The intervention group received a municipality‐based early treatment intervention. Controls (up to five) were matched on age, sex, baseline weight category and calendar year, and received school‐based surveillance consisting of mandatory school health examinations with repeated anthropometric assessments. BMIz trajectories were analysed using mixed effects models with linear splines, adjusting for socioeconomic factors derived from national registries. Among 1274 children and adolescents (216 intervention, 1058 control), 56.9%–58.0% in both groups were living with overweight at baseline. No significant differences in BMIz trajectories were observed over the 4‐year period. Both groups showed an initial decrease in BMIz during the first 6 months (intervention: −0.18; control: −0.28), followed by an increase from 6 to 12 months (intervention: 0.12; control: 0.21) and a plateau from 12 to 48 months (intervention: −0.01; control: −0.03). Municipality‐based interventions did not result in greater long‐term BMIz reductions than matched controls. Both groups had modest short‐term improvements, which may reflect beneficial effects of both the structured interventions and the active school‐based surveillance received by controls. The similar BMIz trajectories suggest system‐level equivalence within the Danish school health system. These findings underscore the need for understanding the mechanisms behind these early changes and how to support long‐term maintenance.

## Summary

- What is already known about this subject?
  ○ Childhood obesity is associated with adverse psychosocial and metabolic outcomes, many of which persist into adulthood.
  ○ Lifestyle interventions are the cornerstone of overweight and obesity prevention and treatment, but sustained long‐term effects are challenging, and evidence from real‐world, municipality‐based settings is limited.
- What this study adds?
  ○ Provides the first long‐term evaluation (up to 4 years) of three different municipality‐based early treatment interventions for childhood overweight and obesity in Denmark.
  ○ Short‐term BMIz reductions were similar in intervention and control groups but diminished over time, suggesting effects of both the interventions and school‐based surveillance with repeated anthropometric assessments.
  ○ Highlights the need for research into the types of support provided outside formal interventions and how these approaches can be optimized.

## 1 Introduction

Overweight and obesity in childhood have increased globally over recent decades, becoming one of the most urgent public health challenges [1, 2]. Although, the overall prevalence has plateaued in Denmark, rates continue to rise among children and adolescents from lower socioeconomic backgrounds, contributing to persistent health inequalities [3, 4, 5]. Children and adolescents with overweight and obesity have increased risks of bullying, social exclusion, stigmatization and low self‐esteem, which may negatively affect academic performance, social development and overall quality of life [6, 7]. Furthermore, children and adolescents are at increased risk of developing physical and psychological comorbidities later in life [8, 9, 10]. Childhood obesity is a complex and multifactorial condition shaped by the interplay of genetic, biological, behavioural, environmental, social and economic factors [1, 11]. The genetic predisposition combined with sedentary behaviour, poor diet quality (e.g., ultra‐processed foods), family dynamics, school environments and broader societal influences all contribute to both its initial development and, importantly for children already affected, to its persistence [11, 12, 13]. These factors often interact in reinforcing cycles, making primary prevention and long‐term secondary prevention/early treatment particularly challenging. As a result, single‐component interventions rarely achieve sustained, meaningful change [10, 11, 14, 15]. Given this complexity and established excess weight at baseline, early treatment strategies are required alongside population‐level prevention. Although recent Cochrane reviews of primary prevention in children aged 6–18 years generally report limited to modest at short‐ to medium‐term effects on BMI and BMI z‐score [16, 17] and long‐term evaluations of school‐based prevention suggest only modest or subgroup‐specific effects over 4–8 years [18, 19], these findings underscore the challenge of achieving durable population‐level impact. In contrast, the present study evaluates early treatment interventions among children already living with overweight or obesity.

Interventions for early treatment should ideally be accessible, feasible, and, when possible, contribute to reducing health disparities [6, 10]. In this context, municipality‐based interventions have gained attention [20]. These approaches involve collaboration among local school nurses, schools, families, and, when necessary, other stakeholders, to promote healthier behaviour and improve well‐being in real‐world settings, aiming existing overweight or obesity and prevent its progression. They are delivered within the child's local environment and often accessible through self‐referral or by the local school nurse. They may be offered in various formats, including family‐based, parent‐only, group‐based, nurse‐led or via telehealth and across diverse settings such as schools, municipality centers or health clinics [21, 22, 23]. Some interventions monitor weight and height regularly, while others adopt a weight‐neutral approach, focusing on promoting healthy behaviours and well‐being without targeting weight change [24].

Despite the growing interest, no evidence‐based consensus exists on the most effective intervention components, for example, mode of delivery, setting or early treatment format [20, 25, 26]. Additionally, many studies are limited by short follow‐up periods, retraining our understanding of the long‐term sustainability of early treatment effects [14, 24, 27, 28].

In Denmark, mandatory school health examinations are conducted at school entry (0–1st), 4–5th and 8–9th grade [29]. Children identified with overweight or obesity may be offered lifestyle interventions through the school nurse or the municipal health center. This study includes three municipalities with three different lifestyle interventions, all targeting children and adolescents with overweight and obesity. The principles underlying these interventions have been previously described [30, 31, 32, 33]. Earlier evaluations in varied settings (e.g., hospital, community) reported mixed results, some short‐term improvements but limited evaluation of long‐term effects, and one was summarized in a report from the Danish Health Authority. However, an evaluation of the long‐term effectiveness of municipal‐based early treatment interventions in a real‐world setting remains to be conducted. Since promoting healthy weight trajectories has been a key aim of these municipal initiatives, combining the interventions with mandatory school health examinations presents a unique opportunity to evaluate their sustained long‐term impact on Body Mass Index z‐score (BMIz) in a real‐world setting. Therefore, the primary aim of this study was to examine changes in BMIz over a 4‐year follow‐up period among children and adolescents with overweight or obesity who have participated in municipality‐based early treatment interventions, compared to matched controls from the same municipalities who received school‐based surveillance with repeated anthropometric assessments. Secondary objectives were to explore differences in early treatment response across the three interventions and to assess whether early treatment effects vary by age, sex and baseline weight category.

## 2 Materials and Methods

### 2.1 Study Design and Setting

This observational cohort study included children and adolescents aged 5–15 years with overweight and obesity, as defined by the International Obesity Task Force (IOTF) cut‐off values [34]. Participants were categorized into two groups during the study period from 1 January 2016 to 31 December 2023: (1) The intervention group included children and adolescents enrolled in municipality‐based lifestyle interventions and (2) a matched control group that consisted of children and adolescents from the municipalities who were not enrolled in these interventions but received school‐based surveillance with repeated anthropometric assessments.

#### 2.1.1 The Intervention Group

Children and adolescents referred to one of three municipality‐based lifestyle interventions in Viborg, Skive, or Holstebro, all located in the Central Denmark Region, were included. Referrals were based on BMI ≥ 25 according to IOTF cut‐offs for overweight [34] and were primarily initiated by municipal health nurses during mandatory school health examinations, but could also occur through general practitioners or self‐referral by the child or parents.

Children were eligible for inclusion if they (1) had a BMI measurement within 6 months before or after the referral (baseline BMI measurement) and (2) had one or more BMI measurement(s) at least 6 months later than baseline BMI measurement (follow‐up BMI measurement). We excluded children who did not have overweight or obesity at referral, had only one BMI measurement, were outside the age range of 5–15 years at referral, or had insufficient time between measurements (> 6 months between referral and baseline or < 6 months between baseline and last visit). The first recorded date of participation in the intervention was defined as the date of referral (index date).

#### 2.1.2 The Control Group

**FIGURE 1:**
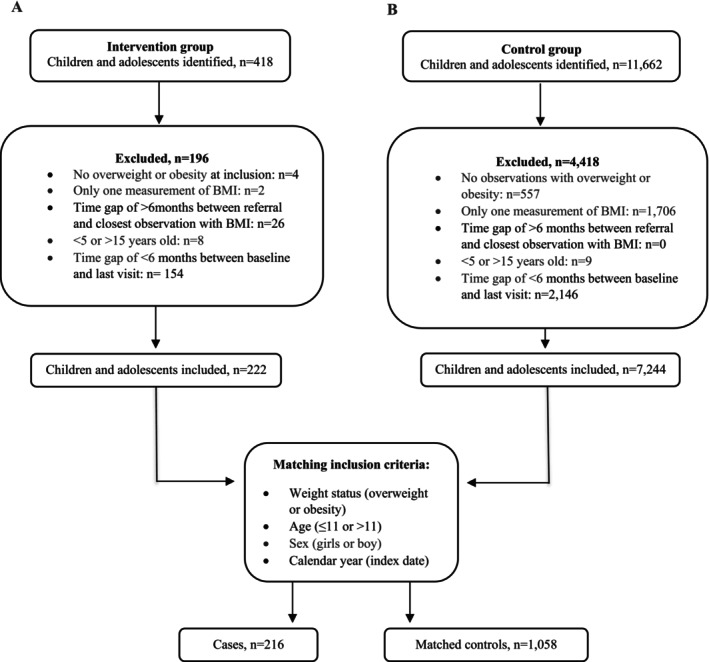
Flowcharts for inclusion, exclusion and matching procedure in which each case was matched with up to five controls and a control could be matched to more than one case, based on 753 unique individuals. Stratified by (A) intervention group, (B) control group, during the study period (1 January 2016–1 January 2024). Exclusions reflect children who did not meet inclusion criteria (e.g., no overweight/obesity at referral, insufficient BMI measurements, or outside age range). Calendar year was defined by the inclusion date (index date), the date intervention participants were registered for a municipality‐based intervention and the date controls had their first recorded BMI indicating overweight or obesity.

For each child in the intervention group, we identified up to five matching controls not enrolled in any of the three interventions. We required controls to have at least one BMI measurement in the calendar year of the index date and another at least 6 months later, as for the intervention child. Children were matched on weight status (overweight or obesity, as classified by the IOTF cut‐off values [34]), age (≤ 11 or > 11 years), sex (girl or boy), and calendar year of index date. Controls were selected from the same calendar year as the corresponding case to control for potential cohort effects. Each control could appear in multiple matched sets (matching with replacement). This approach increases matching quality and statistical efficiency, avoids unnecessary exclusion of eligible controls, and preserved comparability within each risk set. Because the control group represents children participating in school‐based surveillance, including repeated anthropometric assessments, matching with replacement ensures that the characteristics of this active monitored population are consistently represented across comparisons. This procedure was designed to balance the distribution of key variables between the intervention and control groups, reducing confounding factors and enhancing the validity of the comparisons [35]. Six cases were excluded from the analysis because no suitable match was found (Figure 1).

### 2.2 Interventions

The three municipality‐based interventions, mandated by Danish national health policy and delivered through municipal health services, were pragmatic, real‐life programs targeting children and adolescents living with overweight or obesity [36]. All interventions were behavioural and family‐centered aiming to support sustainable lifestyle changes through individualized plans. Core components included guidance on healthy eating, increased physical activity, adequate sleep, reduced screen time and promoting well‐being, enjoyment and self‐esteem. Consultations were conducted with the child and family, while parents were responsible for implementing changes between sessions. The interventions were flexible and adapted to the needs and motivation of each family and follow‐up could extend from several months to multiple years depending on family needs. Participation was provided free of charge to families.

#### 2.2.1 Viborg Municipality

The intervention was based on the multidisciplinary, evidence‐based and non‐randomized ‘Chubby and Healthy’ program from Regional Hospital Viborg and emphasized motivation as a key element [31, 32]. Families received an individualized plan with ‘10 quick goals’ developed collaboratively by the municipal health nurse and the family, focusing on practical behavioural adjustments. Typically, 3–5 consultations were offered initially, with additional sessions available as needed. The program was initially delivered at municipal health centers by municipal health nurses with specialized training and competencies, including a dietician. In 2020, it transitioned to a school‐district model to strengthen interdisciplinary collaboration and was delivered by municipal health nurses who could receive support from two specialized municipal health nurses (previously responsible for the program at the health center). Participation could be offered at any time during the school years (ages 5–16).

#### 2.2.2 Skive Municipality

The intervention was based on the program ‘Small Steps to Sustainable Weight Loss’ recommended by the Danish Health Authority and was tailored to the family's everyday routines [33]. Counselling provided an overview of the family's eating and activity habits and offered practical strategies for sustainable changes, including meal planning and integrating physical activity into daily life. The program typically lasted 6 months and included 4–6 consultations with two additional follow‐up sessions offered annually as needed. Earlier phases involved both the child and parents, while in 2022 phases focused on parents only. The program was delivered at municipal health centers by a health consultant. Participation could be offered at any time during childhood (ages 1–18).

#### 2.2.3 Holstebro Municipality

The intervention was delivered at the municipal health center and was based on the multidisciplinary, evidence‐based and non‐randomized Children's Obesity Clinic's Treatment protocol from Holbæk Hospital [30]. Each program was individualized using medical history and structured interviews to identify necessary lifestyle changes. Consultations were scheduled every 6–8 weeks and typically continued for 12–18 months, with follow‐up appointments included. The intervention was delivered by municipal health nurses with specialized training and competencies. Referrals were typically made through school health nurses. Participation could be offered at any time during the school years (ages 5–16).

### 2.3 Data Sources and Study Variables

#### 2.3.1 Municipality Data

For all children and adolescents included in the study, data were obtained from municipal health databases in Viborg, Skive and Holstebro using data‐capturing systems (i.e., NOVAX and NEXUS) [37]. From databases, unique personal identification numbers (CPR numbers) along with height and weight measurements recorded during interventions and mandatory school health examinations were extracted.

The BMIz was calculated based on weight in kilograms divided by height in meters squared, adjusted for age and sex according to IOTF guidelines [34]. Overweight and obesity were classified using the IOTF cut‐off values [34].

#### 2.3.2 Registry Data

Data on all children and adolescents were linked to their parents by using the unique family ID recorded in the registers of Statistics Denmark (DST). From DST, we had information on maternal pre‐pregnancy BMI (normal weight [BMI < 25.0 kg/m^2^], overweight [25.0 kg/m^2^ ≤ BMI < 30.0 kg/m^2^] or obesity [BMI ≥ 30.0 kg/m^2^]), family type (one‐ or two‐parent family), highest completed household education attained (basic [primary and upper secondary education], medium [vocational education and training], or medium/long [short, medium and long higher education and PhD]), equivalized household income (low [< 33%], medium [33%–66%] and high [> 66%]) and origin of the child (Danish, or non‐Danish [immigrants or descendants of immigrants]).

### 2.4 Ethical Approvals

The study was registered at clinicaltrials.gov (NCT05790174) and in local directories at Aarhus University (ID: 2022‐0367531) and the Central Denmark Region (ID: 1‐16‐02‐487‐21). It was approved by the Central Denmark Region for access to participants records from healthcare databases (ID: 1‐45‐70‐108‐21) and by the Danish Data Protection Agency for the use of registry data from DST.

### 2.5 Statistical Methods

A detailed statistical analysis plan was developed before conducting the analyses (Text S1).

Baseline characteristics of the intervention and control groups were summarized, including sex, age, BMIz, weight category, maternal pre‐pregnancy BMI, family type, highest completed household education, equivalized household income and origin of the child (Table 1). To explore potential selection bias, we compared baseline characteristics of children included in the analysis with those excluded because the interval between baseline and last visit was less than 6 months (*n* = 154) (Table S1).

**TABLE 1:** Characteristics of the study population at baseline.

Variable	Intervention group (*n* = 216)		Control group (*n* = 1058)^a^	
	*n*	Mean (SD)	*n*	Mean (SD)
Age in years, mean (SD)	216	9.56 (2.65)	1058	8.84 (2.92)
BMIz, mean (SD)	216	2.27 (0.60)	1058	2.09 (0.65)
Follow‐up in years, mean (SD)	216	2.42 (1.58)	1058	2.36 (1.67)

	*n*	%^b^	*n*	%^b^
Sex				
Girls	101	46.8	500	47.3
Boys	115	53.2	558	52.7
Weight category				
Overweight	123	56.9	614	58.0
Obesity	93	43.1	444	42.0
Municipalities				
Viborg	128	59.3	448	42.3
Skive	52	24.1	305	28.8
Holstebro	36	16.7	305	28.8
Maternal pre‐pregnancy BMI^c^				
< 25 BMI kg/m^2^	67	31.0	310	29.3
25–30 BMI kg/m^2^	58	26.9	278	26.3
> 30 BMI kg/m^2^	64	29.6	298	28.2
Family type				
One‐parent family	45	20.8	220	20.8
Two‐parent family	171	79.2	827	78.2
Highest completed household^d^ education^a^				
Basic	28	13.0	127	12.0
Short	91	42.1	462	43.7
Medium/long	90	41.7	424	40.1
Equivalized household income^e^				
Low	55	25.5	363	34.3
Medium	96	44.4	373	35.3
High	33	15.3	164	15.5
Origin of the child^f^				
Danish	191	88.4	847	82.6
Non‐Danish	25	11.6	200	18.9
Duration of follow‐up				
Less than a year	51	23.6	—	—
Equal to or more than a year	165	76.4	—	—

Abbreviations: BMIz, body mass index z‐score; SD, standard deviation.

^a^ Based on 753 unique individuals.

^b^ Some columns do not sum to total because of missing values.

^c^ Normal weight (BMI < 25.0 kg/m^2^), overweight (25.0 kg/m^2^ ≤ BMI < 30.0 kg/m^2^) and obesity (BMI ≥ 30.0 kg/m^b^).

^d^ Highest completed household education: (basic [primary and upper secondary education]; short [vocational education and training]; medium/long [short‐, medium‐ and long‐cycle higher education and PhD]).

^e^ Equivalized household income (low [< 33%]; medium [33%–66%]; high [> 66%]).

^f^ Danish or non‐Danish (immigrants or descendants of immigrants).

To assess changes in BMIz over time, we applied two approaches. A mixed effects model with random effects for child ID and match set and included intervention or control status, linear splines with knots at baseline and at 6, 12, 36 and 72 months, and the interaction between intervention status and splines as fixed effects [38]. A linear regression model without splines to estimate overall mean differences in BMIz change between groups. Both models fitted both unadjusted estimates and adjusted estimates for baseline BMIz, family type, household education, equivalized income and origin of the child (Table 2). Maternal pre‐pregnancy BMI was added to the adjusted model in a supplementary analysis due to a high number missing (Table S2). Additionally, stratification of the intervention group by municipality was included in both the unadjusted and adjusted models in a supplementary analysis (Table S3).

**TABLE 2:** Long‐term development in BMI z‐scores (BMIz) among children and adolescents in the intervention and control groups. Yearly changes in BMIz (SD/year) and differences between groups are estimated using mixed‐effects models.

	Change in BMIz per year (95% CI)			
	(a) 0–48 months	(b) 0–6 months	(c) 6–12 months	(d) 12–48 months
	(without linear splines)	(with linear splines)		
(1) Unadjusted model^a^				
Intervention group	−0.01 (−0.02; 0.01)	−0.19 (−0.32; −0.06)	0.07 (−0.06; 0.20)	0.00 (−0.02; 0.02)
Control group	−0.03 (−0.04; −0.02)	−0.25 (−0.38; −0.12)	0.13 (−0.02; 0.27)	−0.02 (−0.03; −0.01)
Difference between groups	0.02 (0.00; 0.04)	0.06 (−0.13; 0.25)	−0.05 (−0.25; 0.14)	0.02 (−0.01; 0.05)
(2) Adjusted model^b^				
Intervention group	0.00 (−0.02; 0.01)	−0.18 (−0.32; −0.03)	0.12 (−0.03; 0.27)	−0.01 (−0.03; 0.02)
Control group	−0.03 (−0.03; −0.02)	−0.28 (−0.43; −0.13)	0.21 (0.05; 0.38)	−0.02 (−0.04; −0.01)
Difference between groups	0.02 (0.00; 0.04)	0.10 (−0.10; 0.31)	−0.09 (−0.31; 0.13)	0.02 (−0.01; 0.05)

Abbreviations: BMIz, body mass index z‐score; CI, confidence interval; SD, standard deviation.

^a^ These models are only adjusted for BMIz at baseline.

^b^ These models are adjusted for BMIz at baseline; sex; family type; highest household education; equivalized household income; and origin of the child.

We examined the stratified effect by age of the child, sex and baseline weight category using unadjusted mixed effects models to explore their potential to modify the intervention effect. For each characteristic, a binary variable was created: age (≤ 11 years [reference] vs. > 11 years), sex (girl vs. boy [reference]) and weight category (overweight vs. obesity [reference]). Interaction was assessed by including the subgroup variable and a three‐way interaction term (e.g., age × intervention × time) in the models. All analyses were performed using Stata/SE 19 (StataCorp LLC, College Station, TX, USA). Two‐sided tests were applied with a significance level of 0.05, and estimates presented with 95% confidence intervals (CI).

## 3 Results

A total of 1274 children and adolescents were included in the study: 216 (56.9% with overweight) in the intervention group and 1058 (58.0% with overweight) in the control group (Table 1). At baseline, the intervention group included participants who were older, had a higher BMIz, higher levels of completed household education and a greater proportion of Danish origin compared to those in the control group. Within the intervention group, most participants were from Viborg municipality (59.3%), followed by Skive (24.1%) and Holstebro (16.7%) (Table 1). Children and adolescents who were excluded due to a time gap of less than 6 months between referral and last intervention visit (*n*: 152) were older compared with those who remained in the intervention group (Table S1).

In the adjusted model, no significant differences were observed between the intervention and control groups over the 4 year period. During the first 6 months, both groups reduced BMIz (intervention group: −0.18 vs. control group: −0.28; difference: 0.10 SD/years, 95% CI: −0.10, 0.31). From 6 to 12 months, both groups increased BMIz (intervention group: 0.12 vs. control group: 0.21; difference: −0.09 SD/years, 95% CI: −0.32, 0.13) followed by a minor decrease in BMIz from 12 to 48 months (intervention group: −0.01 vs. control group: −0.03; difference: 0.02 SD/years, 95% CI: −0.01, 0.05) (Table 2). In analyses without splines across time points, the adjusted model showed no meaningful change in BMIz for (intervention group: 0.00 vs. control group: −0.03; differences: 0.03 SD/years, 95% CI: 0.00, 0.04), similar to the unadjusted model (Table 2). Similar results were observed in the adjusted analysis including maternal pre‐pregnancy BMI (Table S2), and when stratifying the intervention group by municipality (Table S3).

Stratified analyses revealed group‐specific trends in BMIz change over time. Among children and adolescents aged > 11 years, BMIz decreased in both the intervention (−0.08 SD/year; 95% CI: −0.12; −0.04) and the control groups (−0.13 SD/year; 95% CI: −0.15; −0.10) (Table 3). Effect modification analysis using three‐way interaction terms indicated that age did not modify the intervention effect (0.03 SD/year; −0.03; 0.08).

**TABLE 3:** Yearly change in BMIz (SD/year) in the intervention and control groups stratified by age, sex and weight category at baseline (unadjusted mixed‐effects models).

	Change in BMIz per year (95% CI)	
	Intervention group	Control group
Age		
≤ 11	0.00 (−0.01; 0.02)	−0.02 (−0.03; −0.01)
> 11	−0.08 (−0.12; −0.04)	−0.13 (−0.15; −0.10)
Sex		
Girls	0.04 (0.02; 0.06)	−0.02 (−0.03; −0.01)
Boys	−0.06 (−0.08; −0.04)	−0.04 (−0.05; −0.03)
Weight category		
Overweight	0.00 (−0.01; 0.02)	−0.02 (−0.03; −0.01)
Obesity	−0.03 (−0.05; −0.01)	−0.05 (−0.06; −0.03)

Abbreviations: BMIz, body mass index z‐score; CI, confidence interval; SD, standard deviation.

In the sex‐stratified analysis, BMIz increased among girls in the intervention group (0.04 SD/year; 95% CI: 0.02; 0.06), while boys had a decrease (−0.06 SD/year; 95% CI: −0.08; −0.04). In the control group, BMIz decreased slightly for both sexes (Table 3). The interaction with sex suggests a differential intervention effect (0.07 SD/year; CI: 0.04; 0.11), indicating a less favourable response among girls in the intervention group.

When stratified by baseline weight category, BMIz decreased among children and adolescents living with obesity in the intervention group, whereas in the control group BMIz decreased in both weight categories (Table 3). The interaction indicated comparable intervention effects among children living with overweight or obesity (−0.1 SD/years; −0.04; 0.03).

## 4 Discussion

### 4.1 Key Results

This study examined the long‐term effect of three municipality‐based interventions on BMIz among children and adolescents living with overweight or obesity, compared with a matched control group who received school‐based surveillance with repeated anthropometric assessments. Over a follow‐up period of up to four years, no significant differences in BMIz trajectories were observed between intervention and control groups. Both groups showed an initial decrease in BMIz during the first 6 months, followed by an increase from 6 to 12 months and a plateau from 12 to 48 months. The effect modification analyses revealed that girls in the intervention group had more pronounced increases in BMIz over time compared to boys. In contrast, age and baseline weight category did not modify the intervention effect.

### 4.2 Interpretation of Results

The initial decrease in BMIz after 6 months and the subsequent absence of a long‐term effect (≥ 48 months) aligns with prior findings in municipality‐ or community‐based early treatment interventions [39, 40]. These studies primarily included children with obesity, whereas our study included both of children and adolescents, and was dominated by participants with overweight, highlighting an evidence gap for this subgroup. In this study, more than half of the intervention participants were classified with overweight rather than obesity. This suggests that municipality‐based programs may primarily aim to prevent progression from overweight to obesity rather than achieve substantial weight reduction. This focus may be meaningful, given that weight status tracks into adulthood, obesity significantly increases the risk of comorbidities [8, 9] and prevalence of obesity is expected to rise substantially by 2025 [2]. Interventions involving children with overweight are typically population‐based programs targeting all weight categories, which often show limited effects because outcomes are diluted across mixed weight status groups [10]. Evidence also suggests that behaviour and weight changes are generally more achievable in children with obesity than in those with overweight [41, 42]. However, no meaningful effect modification by baseline weight category was observed, indicating broadly similar effects across these groups.

The early reduction in BMIz observed in both the intervention and control groups likely reflects system‐level influences, particularly the comprehensive school‐based surveillance system in which all children participated. This system includes repeated anthropometric assessments and follow‐up, which may increase awareness among families and initiate short‐term behaviour changes, regardless of participation in a structured early treatment intervention. These context factors may help explain the parallel early improvements and underscore the importance of considering broader system‐level mechanisms when interpreting early BMIz changes.

Although the multicomponent approaches in the structured early treatment interventions combining recommendations of physical activity, diet, behavioural strategies, family involvement and delivery across school or community settings, align with previous evidence [14, 43, 44], it may not have been sufficient to produce sustained BMIz reductions. It is also possible that the intervention helped prevent further weight gain among participants with obesity or other high‐risk characteristics, which would still represent a clinically meaningful outcome. It is important to acknowledge that BMIz, while widely used in paediatric research, has limitations. It does not distinguish fat and lean mass or capture improvements in physical activity, well‐being and health behaviours, which are clinically relevant and may contribute to long‐term health even if not reflected in BMIz trajectories [45].

Addressing childhood overweight and obesity ultimately requires system‐level strategies beyond individual behaviour change and isolated intervention programs, including supportive policies, communities and environments that shape food access, physical activity opportunities and health behaviours [13, 42, 46]. Contemporary public health frameworks emphasize that obesity develops within broader obesogenic environments influenced by food systems, economic conditions, marketing and social norms. These structural drivers inherently limit the potential for individual‐level lifestyle interventions to archive lasting change. Our findings align with the growing consensus that durable impact will require coordinated primary prevention efforts—along with clinical and municipal approaches for early treatment. Relevant examples include policies that improve food environments, regulate marketing and promote settings supporting more physical activity [13, 47].

Most subgroup differences were small and likely of limited clinical relevance. However, the less favourable trajectory among girls may reflect biological factors such as pubertal development [48]. In the intervention group, almost 25% (*n* = 24) of girls were older than 11 years (Table S4), and pubertal changes during follow‐up may have contributed to the observed pattern. A recent systematic review reported no consistency in sex‐specific response in lifestyle interventions among adolescents with overweight and obesity [49]. Further, no meaningful effect modification by age was observed.

### 4.3 Strengths and Limitations

One key strength of this study is the linkage of clinical data with nationwide Danish registries, which enabled long‐term follow‐up the in real‐world settings. This approach minimized risks of differential loss to follow‐up, selection bias and recall bias. Furthermore, the school‐based surveillance system, as required by the Danish Health Authority, ensured standardized and high‐quality measurements of height and weight across both intervention and control groups [29]. These examinations also enabled the matched control group within the same municipalities, with up to five controls per intervention participants, improving the robustness of comparisons. In addition, registry‐based socioeconomic and demographic data were collected at baseline for both groups, ensuring a high degree of comparability due to the comprehensive coverage and validated methods used in the national registers. Finally, the extended follow‐up period of up to 4 years enabled the assessment of both short‐ and long‐term intervention effects.

The study has some limitations. First, up to a 6‐month gap between referral and the first BMI measurement, may have influenced early BMIz changes, however, this is unlikely to affect interpretation of long‐term effects. Second, despite the matching strategy and statistical adjustment, residual confounding may persist. One potential source is the imprecise definition of baseline BMIz, because the timing of these examinations varies, baseline values may capture short‐term fluctuations rather than a stable underlying measure. In addition, unmeasured factors such as health literacy, family motivation and readiness for behaviour change, or prior health promotion support may differ between groups and influence responsiveness to intervention. As with any observational design, such unmeasured or imperfectly measured factors may influence outcomes. Additionally, six children could not be matched and were excluded (Figure 1), which may introduce minor selection bias if these children differed systematically from those included in the analysis. Third, referral based on IOTF cut‐offs [34] may have led to less complex cases among controls, who, despite meeting referral criteria, did not receive the intervention. Some may have declined, not attended, or accessed other health services. Fourth, mandatory school health examinations occur up to three times during a child's school year. Among controls, 34.1% (*n* = 361) had more than five follow‐up visits during the 4 years period (Table S5), suggesting that some may have undergone additional examinations or received health‐related support from school health nurses, potentially influencing BMIz trajectories. These findings should be interpreted in the context that the control group received school‐based surveillance with repeated anthropometric assessments, meaning the comparator was an active follow‐up system rather than a minimal‐care group. Further, more than 7000 controls (Figure 1) met criteria for intervention participation, raising questions about referral practices and resource constraints. If children living with overweight or obesity received additional support outside the formal intervention, isolating the intervention effect becomes difficult. Fifth, we were unable to account for individual weight trajectories prior to baseline, which limits interpretation of subsequent changes and whether the intervention may have prevented further weight gain. Sixth, a large proportion of the participants were from Viborg municipality (59.3%), compared to Skive (24.1%) and Holstebro (16.7%). This uneven distribution, together with the modest overall sample size, limited statistical power to evaluate municipal‐specific effects. We stratified analyses by municipality, but estimates were imprecise and did not support firm conclusions regarding differences in outcomes. Although the intervention programs differed in specific components and modes of delivery, this heterogeneity reflects real‐world variation in how a common intervention approach was implemented across municipalities, rather than representing fundamentally distinct intervention types. Lastly, the intervention group had higher household education levels and socioeconomic factors influence health behaviours and risk of obesity, potentially confounding the results. Although household education and income were adjusted for, residual confounding is an inherent limitation of observational designs and cannot be ruled out. This remains important because such factors may have influenced baseline behaviours or responsiveness to intervention, even if overall group differences were small. Given the matched design, stratification by socioeconomic subgroups was not feasible, limiting assessment of differential effects. Nonetheless, the null findings were robust to extensive adjustment for socioeconomic indicators, suggesting that substantial unmeasured confounding would be required to materially change the conclusions.

Future programs may benefit from closer integration between municipal early treatment initiatives and school health services, greater attention to socioeconomic disparities, and the inclusion of outcomes beyond BMIz such as health behaviour and psychosocial well‐being.

## 5 Conclusion

Municipality‐based early treatment interventions did not result in greater long‐term BMIz reductions compared to the matched controls receiving school‐based surveillance with repeated anthropometric assessments. The similar BMIz trajectories across groups suggest system‐level equivalence within the Danish school health system, where ongoing monitoring and repeated anthropometric assessment are routine provided. Both groups experienced short‐term improvements, likely reflecting effects of both targeted municipal early treatment interventions and existing school health examinations. These findings underscore the importance of interpreting early treatment efforts for childhood overweight and obesity within its broader system‐level context. Larger studies are needed to determine which early treatment approaches work best for which children and how early improvements can be translated into sustained, long‐term benefits.

## Author Contributions

M.F., J.N.Ø. and J.M.B. conceived the study hypothesis. M.F. prepared the dataset and drafted the initial statistical analysis plan, which all co‐authors critically reviewed, contributed to, and revised. M.F. handled the matching procedures; H.S. suggested and supervised the matching strategy. M.F. did the statistical analysis. M.F. wrote the initial draft, with critical revisions from all co‐authors. M.F. finalized the draft, and all authors reviewed and approved the final version.

## Funding

The study was supported by a public grant from the Danish Regions through ‘The Joint Grant for Prevention’ (grant no.: R201‐A4369 (2022)) and by the Steno Diabetes Center Aarhus, which is partially funded by an unrestricted grant from the Novo Nordisk Foundation. The content of this article reflects the views of the authors alone. The funders had no role in study design, data collection and analysis, manuscript preparation, or the decision to publish.

## Conflicts of Interest

The authors declare no conflicts of interest.

## Supporting information


**Table S1:** Baseline characteristics of the study population of included and excluded intervention group.
**Table S2:** Long‐term BMIz development among children and adolescents in the intervention and control group, with additional adjustment for pre‐pregnancy BMI. Yearly changes in BMIz (SD/year) and differences between groups are estimated using mixed‐effects models.
**Table S3:** Long‐term BMIz development among children and adolescents between the three intervention and control groups, stratified by municipality. Yearly changes in BMIz (SD/year) and differences between groups are estimated using mixed‐effects models.
**Table S4:** Distribution of girls by pubertal status (≤ 11 vs. > 11 years) in intervention and control group.
**Table S5:** Number of follow‐up visits after baseline in intervention and control group.

## Data Availability

The data used in this study include sensitive personal health information collected from municipal health databases. Due to Danish data protection regulations (GDPR), these data cannot be shared publicly. Access to the national registry data used for linkage and socioeconomic variables is governed by Statistics Denmark and the Danish Health Data Authority. Researchers interested in accessing similar registry data may apply through the appropriate national channels. Access to municipal health data is subject to local institutional approvals and is not publicly available.
