# Supplementary material for: Municipality‐Based Lifestyle Intervention of Childhood Overweight and Obesity: 4‐Years Follow‐Up on BMI Trajectories
Source: Clin Obes. 2026 May 5;16:e70082. doi: 10.1111/cob.70082 (PMC13143494; doi:10.1111/cob.70082)
Supplement: Supplementary file 1 — Table S1: Baseline characteristics of the study population of included and excluded intervention group. Table S2: Long‐term BMIz development among children and adolescents in the intervention and control group, with additional adjustment for pre‐pregnancy BMI. Yearly changes in BMIz (SD/year) and differences between groups are estimated using mixed‐effects models. Table S3: Long‐term BMIz development among children and adolescents between the three intervention and control groups, stratified by municipality. Yearly changes in BMIz (SD/year) and differences between groups are estimated using mixed‐effects models. Table S4: Distribution of girls by pubertal status (≤ 11 vs. > 11 years) in intervention and control group. Table S5: Number of follow‐up visits after baseline in intervention and control group. [file COB-16-e70082-s001.pdf]

Supplementary material

Title: Municipality-based lifestyle intervention of childhood overweight and obesity: 4-years follow-up on BMI trajectories

Authors:

Mette Fogh, ORCID 0000-0002-5163-77281,2

Jane Nautrup Østergaard, ORCID 0000-0002-0627-1237[1,3]

Helene Kirkegaard, ORCID 0000-0001-6877-916X[1]

Eeva-Liisa Røssell, ORCID 0000-0001-6423-383X [1]

Helle Terkildsen Maindal, ORCID 0000-0003-0525-7254[4]

Gunnar Toft, ORCID 0000-0002-7542-6853[1,4]

Henrik Støvring, ORCID 0000-0002-5821-2351[1,5,6]

Jens Meldgaard Bruun, ORCID 0000-0001-9937-5467[1,2,7]

Affiliations:

[1]Steno Diabetes Center Aarhus, Aarhus University Hospital, DK-8200 Aarhus N, Denmark

[2]Department of Clinical Medicine, Aarhus University, DK-8200 Aarhus N, Denmark

[3]National Institute of Public Health, University of Southern Denmark, Copenhagen K, Denmark.

[4]Department of Public Health, Aarhus University, DK-8000 Aarhus C, Denmark

[5]Department of Biomedicine, Aarhus University, DK-8000 Aarhus C, Denmark

[6]Clinical Pharmacology, Pharmacy and Environmental Medicine, University of Southern Denmark, DK-5230 Odense M, Denmark

[7]Danish National Center for Obesity, DK-8200 Aarhus N, Denmark

Correspondence: Mette Fogh; Steno Diabetes Center Aarhus, Aarhus University Hospital, Palle Juul-Jensen Blvd. 11, entrance A, 8200 Aarhus N, Denmark. E-mail: mefogh@rm.dk

Supplementary material

Table of contents

Supporting tables **3**
Table S1 3
Table S2 4
Table S3 4
Table S4 4
Table S5 4
Supporting text **6**
Text S1. Statistical analysis plan 6

Supplementary material

Supporting tables

Table S1 Baseline characteristics of the study population of included and excluded intervention group

	Intervention group (n=216)		Excluded – time gap <6 months (n=154)	
**Variable**	**n**	**Mean (SD)**	**n**	**Mean (SD)**
Age (years), mean (SD)	216	9.56 (2.65)	154	10.77 (2.70)
BMIz, mean (SD)	216	2.27 (0.60)	154	2.26 (0.55)
Follow-up (year), mean (SD)	216	2.42 (1.58)	-	-
	**n**	**%**[1]	**n**	**%**[1]
Girls	101	46.8	68	44.2
Weight category				
Overweight	123	56.9	89	57.8
Obesity	93	43.1	65	42.2
Municipalities				
Viborg	128	59.3	126	81.8
Skive	52	24.1	12	7.8
Holstebro	36	16.7	16	10.4
Maternal pre-pregnancy BMI[2]				
< 25 BMI kg/m^2	67	31.0	50	32.5
25-30 BMI kg/m^2	58	26.9	37	24.0
> 30 BMI kg/m^2	64	29.6	52	33.8
Family type				
One-parent family	45	20.8	27	17.5
Two-parent family	171	79.2	120	77.9
Highest completed household education[3]				
Basic	28	13.0	11	7.1
Short	91	42.1	41	26.6
Medium/long	90	41.7	49	31.8
Equivalized household income[4]				
Low	55	25.5	16	10.4
Medium	96	44.4	25	16.2
High	33	15.3	5	3.3
Origin of the child[5]				
Danish	191	88.4	132	85.7
Non-Danish	25	11.6	15	9.7

Abbreviations: SD, standard deviation; BMIz, body mass index z-score
[1]Some columns do not sum to total because of missing values.
[2]Normal weight (BMI < 25.0 kg/m2), overweight (25.0 kg/m^2 ≤ BMI < 30.0 kg/m^2), and obesity (BMI ≥ 30.0 kg/m^2).
[3]Highest completed household education: (basic [primary and upper secondary education]; short [vocational education and training]; medium/long [short-, medium- and long-cycle higher education and PhD]).
[4]Equivalized household income (low [< 33 %]; medium [33-66 %]; high [> 66 %]).
[5]Danish or non-Danish (immigrants or descendants of immigrants)

Supplementary material

Table S2 Long-term BMIz development among children and adolescents in the intervention and control group, with additional adjustment for pre-pregnancy BMI. Yearly changes in BMIz (SD/year) and differences between groups are estimated using mixed-effects models.

	a) 0 to 48 months	b) 0 to 6 months	c) 6 to 12 months	d) 12 to 48 months
	(without linear splines)	(with linear splines)		
Adjusted model[1]				
Intervention group	-0.01 (-0.02; 0.01)	-0.17 (-0.32;-0.01)	0.10 (-0.06;0.26)	-0.01 (-0.03;0.10)
Control group	-0.03 (-0.03;-0.02)	-0.32 (-0.49; -0.16)	0.24 (0.06;0.43)	-0.02 (-0.04;-0.01)
Difference between groups	-0.02 (-0.04; 0.00)	-0.16 (-0.39;0.07)	0.15 (-0.10;0.39)	-0.02 (-0.05;0.01)

Abbreviations: BMIz, body mass index z-score

[1]These models are adjusted for BMIz at baseline; sex; family type; highest household education; equivalized household income; and origin of the child.

Table S3 Long-term BMIz development among children and adolescents between the three intervention and control groups, stratified by municipality. Yearly changes in BMIz (SD/year) and differences between groups are estimated using mixed-effects models.

	a) 0 to 48 months	b) 0 to 6 months	c) 6 to 12 months	d) 12 to 48 months
	(without linear splines)	(with linear splines)		
1) Unadjusted model[1]				
Intervention group – Viborg	-0.01 (-0.03; 0.00)	-0.05 (-0.24;0.14)	0.09 (-0.12;0.31)	-0.03 (-0.07;0.00)
Intervention group – Skive	0.00 (-0.02; 0.03)	-0.28 (-0.53;-0.03)	0.06 (-0.17;0.30)	0.03 (-0.02;0.07)
Intervention group – Holstebro	-0.02 (-0.07;0.04)	-0.39 (-0.66;-0.11)	0.01 (-0.25;0.28)	0.09 (-0.01;0.19)
Control group	-0.03 (-0.04; -0.02)	-0.25 (-0.38;-0.12)	0.13 (-0.02;0.27)	-0.02 (-0.03;-0.01)
2) Adjusted model[2]				
Intervention group – Viborg	-0.01 (-0.04; 0.01)	-0.07 (-0.30;0.16)	0.18 (-0.08;0.44)	-0.04 (-0.08;0.00)
Intervention group – Skive	0.00 (-0.03; 0.03)	-0.21 (-0.48;0.07)	0.06 (-0.26;0.26)	0.03 (-0.01;0.07)
Intervention group – Holstebro	0.01 (-0.05;0.07)	-0.37 (-0.74;-0.01)	0.08 (-0.25;0.42)	0.07 (-0.03;0.18)
Control group	-0.02 (-0.03; -0.02)	-0.32 (-0.49;-0.16)	0.24 (0.06;0.43)	-0.02 (-0.04;-0.01)

Abbreviations: BMIz, body mass index z-score.

[1]These models are only adjusted for BMIz at baseline

[2]These models are adjusted for BMIz at baseline; sex; family type; highest household education; equivalized household income; and origin of the child.

Table S4 Distribution of girls by pubertal status (≤11 vs. >11 years) in intervention and control group

	**Intervention group (n=101)**		**Control group (n=500)**	
**Variable**	**n**	**%**	**n**	**%**
Age				
≤11	77	76.2	385	77.0
>11	24	23.8	115	23.0

Table S5 Number of follow-up visits after baseline in intervention and control group

	**Intervention group (n=216)**		**Control group (n=1,058)**	
**Number of follow-up visits**	**n**	**%**	**n**	**%**
2	36	16.7	353	33.4
3	33	15.3	125	11.8

4	32	14.8	219	20.7
5+	115	53.2	361	34.1

Supporting text

Text S1. Statistical analysis plan

Statistical analysis plan

Municipality-based lifestyle intervention of childhood overweight and obesity: 4-years follow-up from a Danish cohort

SUGGESTED AUTHORS:

Mette Fogh, Jane Nautrup Østergaard, Helene Kirkegaard, Eeva-Liisa Røssell, Gunnar Vase Toft, Helle Terkildsen Maindal, Henrik Støvring, Jens Meldgaard Bruun

PURPOSE:

This statistical analysis plan outlines the background and rationale for the study, along with key aspects of data preparation and analysis. The plan was developed and approved by all co-authors before the initiation of any statistical analyses. It will be submitted as supplementary material alongside the manuscript.

BACKGROUND:

Overweight and obesity in childhood have increased globally over recent decades, becoming one of the most urgent public health challenges worldwide (1, 2). Although the overall prevalence has plateaued in Denmark, rates continue to rise among children and adolescents from lower socioeconomic backgrounds, contributing to persistent health inequalities (3, 4). Children and adolescents with overweight and obesity have increased risks of bullying, social exclusion, stigmatization, and low self-esteem, which may negatively affect academic performance, social development, and overall quality of life (5, 6). Furthermore, they are at increased risk of developing physical and psychological comorbidities later in life (7-9).

Childhood obesity is a complex and multifactorial condition shaped by the interplay of genetic, biological, behavioral, environmental, social, and economic factors (2, 10). The genetic predisposition combined with sedentary behavior, poor diet quality, family dynamics, school environments, and broader societal influences all play a role in its development and persistence (10, 11). These factors often interact in reinforcing cycles, making prevention and treatment particularly challenging. As a result, single-component interventions rarely achieve long-term, meaningful change (10, 12, 13). Effective interventions must be accessible, feasible, and capable of reducing health disparities (5, 9).

In this context, municipality-based interventions have gained attention (14). These approaches involve collaboration among local school nurses, schools, families, and, when necessary, other stakeholders, to promote healthier behavior and improve well-being in real-world settings. Delivered within the child's local environment and often accessible through self-referral or by the local school nurse, municipality-based interventions aim to strengthen the municipal response to childhood overweight and obesity by improving both the availability and accessibility of supportive health-promoting services (15-17). They may be delivered in various formats, including family-based, parent-only, group-based, nurse-led or via telehealth, and across diverse settings such as schools, municipality centers, or health clinics (18-20). Some interventions include regular weight and height monitoring as a part of follow-up. In contrast, others adopt a

weight-neutral approach, focusing on promoting healthy behaviors and well-being without explicitly aiming for weight change (21).
Despite the growing interest, no evidence-based consensus exists on the most effective intervention components, e.g., mode of delivery, setting, or treatment format (14, 22-24). Additionally, many studies are limited by short follow-up periods, limiting our understanding of the long-term sustainability of treatment effects (12, 21, 25, 26).
In Denmark, mandatory school health examinations are conducted at school entry (0-1st), 4-5th (recommended), and 8-9th grades (27). If a child is identified with overweight or obesity, they may be offered lifestyle intervention through the school or the municipal health center. Three municipalities with three different lifestyle interventions are included in this study, all three targeting children and adolescents with overweight and obesity. The three interventions have previously been evaluated in other contexts or are recommended by the Danish Health Authority (17, 28, 29), but they have not yet been systematically assessed in terms of long-term effectiveness on weight development. Since promoting healthy weight trajectories has been a key aim of these municipal initiatives, combining the interventions with routine school health examinations presents a unique opportunity to evaluate their sustained impact on body mass index z-score (BMIz) over time, using matched control groups from the same municipalities.

OBJECTIVE:
The primary objective of this study is to examine changes in BMIz over a four-year follow-up period among children and adolescents with overweight or obesity who have participated in municipality-based overweight and obesity interventions, compared to matched controls from the same municipalities.

Secondary objectives:
- Explore differences in treatment response across the three municipality-based lifestyle interventions
- Assess whether treatment effects vary by age, sex, and baseline weight category

MATERIALS:
Study population
This study includes children and adolescents aged 5 to 15 years who have a measurement of overweight or obesity, as defined by the International Obesity Task Force (IOTF) cut-off values (30). Participants are categorized into two groups during the study period from 1 January 2016 to 1 January 2024.
1. An *intervention group*, consisting of children and adolescents who participated in a municipality-based lifestyle intervention
2. A *control group*, consisting of children and adolescents who did not receive this treatment

The intervention group consists of children and adolescents referred to one of three municipality-based lifestyle interventions offered in the municipalities of Viborg, Skive, and Holstebro, all located in the Central Denmark Region. Children were eligible for inclusion if they 1) were referred to and participated in one of the three municipality-based lifestyle interventions, 2) had a weight measurement within six months before or after the referral (baseline weight measurement) and 3) had another weight measurement at least six months later (follow-up weight measurement). We excluded children who did not have overweight or obesity at referral, had only one BMI measurement, were outside the

age range of 5-15 years at inclusion, or had insufficient time between measurements (≤6 months between baseline and inclusion, or >6 months between inclusion and last visit). The first recorded date of participation was defined as the date of inclusion (index date).

The control group, for each child in the intervention group, we identified up to five matching controls with no enrollment in any of the three interventions. Controls were required to have at least one BMI measurement in the calendar year of the index date and another at least six months later, as for the intervention child. Children were matched on:

- Weight status (overweight or obesity, as defined by the IOTF)
- Age (≤11 or >11)
- Sex (girl or boy)
- Municipality (Viborg, Skive, or Holstebro)
- Inclusion calendar year

To maximize statistical efficiency and minimize bias, each control can be included multiple times. If no appropriate match is found for a given intervention participant, that participant is excluded from the analysis.

Outcome

Primary outcomes: Changes in BMIz from the time of inclusion and up to four years of follow-up, comparing children and adolescents in the intervention group with those in the matched control group.

DATA SOURCES, STUDY VARIABLES, AND COVARIABLES

Municipality data

For all children and adolescents included in the study data were obtained from municipal health databases in Viborg, Skive, and Holstebro using NOVAX, a data-capturing system used by school health nurses. From NOVAX, we extracted personal identification numbers (CPR numbers), along with height and weight measurements recorded during mandatory school health examinations. For children in the intervention group in Skive, participation data was obtained from NEXUS.

The BMIz was calculated based on weight in kilograms divided by height in meters squared, adjusted for age and sex according to the International Obesity Task Force (IOTF) guidelines (30). Overweight and obesity were classified using the IOTF cut-off values (30).

The study was approved by the Regional Secretariat of the Central Denmark Region for the disclosure of information from patient records (approval no. 1-45-70-108-21).

Registry data

The included children and adolescents will be linked to their parents by using the unique family ID recorded in the registers of Statistics Denmark (DST) at inclusion in intervention group or control group. Information on maternal pre-pregnancy BMI will be derived from the Danish Medical Birth Register (31). Maternal pre-pregnancy BMI is collected at the first antenatal care visit and categorized into three groups according to WHO classification: normal weight (18.5 kg/m2 ≤ BMI < 25.0 kg/m2), overweight (25.0 kg/m2 ≤ BMI < 30.0 kg/m2), and obesity (BMI ≥ 30.0 kg/m2)(32).

Supplementary material

Family type will be defined as children and adolescents living in a two-parent family or one-parent family as recorded in the Danish Population Register of DST. The highest completed household education attained will be grouped into three categories: *basic* (primary and upper secondary education), *medium* (vocational education and training), and *medium/long* (short, medium and long higher education and PhD) as recorded in the Danish Education Register of DST (33-35). Equivalised household income (Danish Kroner (DKK)) was adjusted for taxes, alimony, and the number of family members and grouped into tertiles; *low* (< 33 %), *medium* (33-66 %), and *high* (> 66%) income as recorded in the Danish Household Register of DST (36, 37). The child's country of origin was defined as Danish or non-Danish (immigrants or descendants of immigrants) origin as recorded in the Immigration Register of DST.

STATISTICAL METHODS:

All statistical analyses will be conducted using Stata/SE 18 (StataCorp LP, College Station, Texas, USA). All tests will be two-sided with a significance level of 0.05, and results reported with 95% confidence intervals.

Baseline characteristics of the study population

We will describe baseline characteristics for the intervention and control groups of sex, age, BMIz, weight category, maternal pre-pregnancy BMI, family type, highest completed household education, equivalised household income, and the child's country of origin (Table 1).

Long-term weight development in the intervention versus control group

To examine BMIz trajectories over time, we apply a mixed effects model with linear splines with knot points placed at date of inclusion, and at 6, 12, and 48 months. Analyses will be performed both unadjusted and adjusted for baseline BMIz, maternal pre-pregnancy BMI, family type, household education, equivalised income, and country of origin (Table 2).

Effect modification by subgroups

We will conduct stratified analyses by age group, sex, and weight category (Table 3) using a mixed effects models to explore their potential to modify the intervention effect. Effect modification will be formally assessed by including each subgroup variable (e.g. ≤11 (reference (ref)) vs. >11) and a corresponding three-way interaction term (e.g. age x intervention x years) in the models. For each characteristic, the largest subgroup serves as the reference category.

Supplementary material

RESULTS

This section presents the templates of figures and tables related to statistical analyses planned a priori. Some additional sensitivity analyses may be added subsequently.

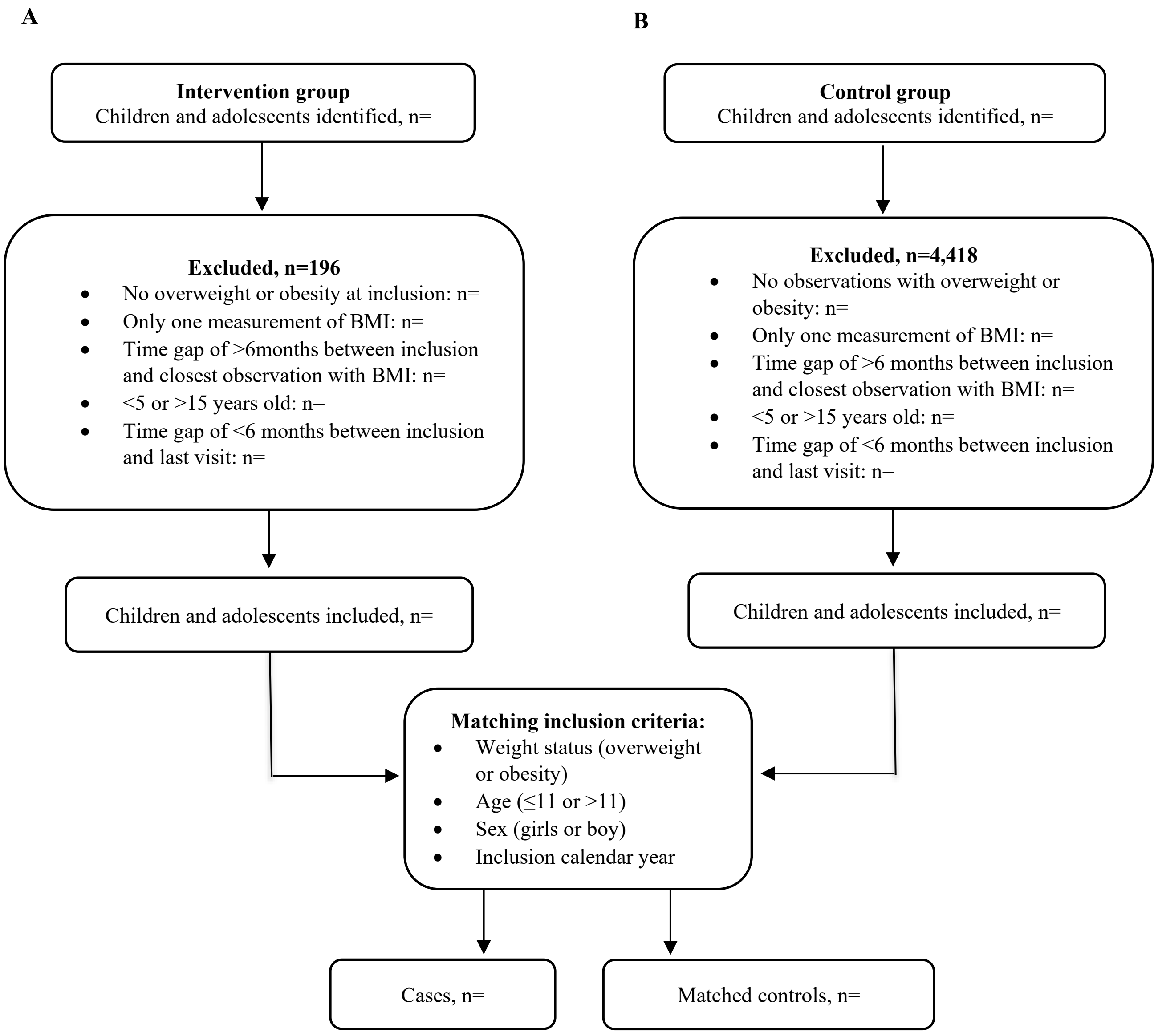

Figure 1 Flowcharts for inclusion, exclusion, and matching procedure in which each case is matched with up to five controls, stratified by A) intervention group, B) control group, during the study period (1 January 2016–1 January 2024). Exclusions reflect children who did not meet inclusion criteria (e.g., no overweight/obesity at referral, insufficient BMI measurements, or outside age range). The index year is defined as the calendar year in which the intervention participants were registered for inclusion in a municipality-based intervention and controls had their first recorded BMI indicating overweight or obesity.

Supplementary material

Table 1 Characteristics of the study population at baseline.

	**Intervention group**	**Control group**
**Variable**	n	n
Girls, n (%)		
Age, years (Mean (SD))*		
BMIz (Mean (SD))*		
Follow-up, year (Mean (SD))*		
Weight category, n (%)		
Overweight		
Obesity		
Municipalities, n (%)		
Viborg		
Skive		
Holstebro		
Maternal pre-pregnancy BMI, n (%)		
< 25 BMI kg/m^2		
25-30 BMI kg/m^2		
> 30 BMI kg/m^2		
Family type, n (%)		
One-parent family		
Two-parent family		
Highest completed household[1] education[1], n (%)		
Basic		
Short		
Medium/long		
Equivalized household income[2], n (%)		
Low		
Medium		
High		
Child's country of origin, n (%)		
Danish		
Non-Danish		

Abbreviations: BMIz, body mass index z-score

[1]Highest completed household education: (basic [primary and upper secondary education]; short [vocational education and training]; medium/long [short-, medium- and long-cycle higher education and PhD]).

[2]Equivalized household income (low [< 33 %]; medium [33-66 %]; high [> 66 %]).

*If data is not normally distributed, the median will be reported.

Supplementary material

Table 2 Long-term development in BMI z-scores (BMIz) among children and adolescents in the intervention and control groups. Yearly changes in BMIz (expressed as SD/year) and differences between groups are estimated using mixed-effects models.

	**a) Change in BMIz per year (without splines)**	**b) 0 to 6 months**	**c) 6 to 12 months**	**d) 12 to 48 months**
		Changes in BMIz per year (95% CI) (with linear splines)		
*1) Unadjusted model**				
Intervention group				
Control group				
Difference between groups				
2) Adjusted model#				
Intervention group				
Control group				
Difference between groups				

Abbreviations: BMIz, body mass index z-score

[1]) Two levels of adjustments are applied: 1) adjustments for BMIz at inclusion and 2) additional adjustments for BMIz at inclusion, family type, equivalized household income, highest completed household education, and child's country of origin. Differences in BMIz per year were analyzed across the following time periods: a) all available observations, b) 0 to 6 months, c) 6 months to 12 months, and d) 12 months to 48 months

*These models are only adjusted for BMIz at inclusion

#These models are adjusted for BMIz at inclusion; sex; family type; highest household education; equivalized household income; and child's country of origin.

Supplementary material

Table 3 Annual change in BMIz (SD/year) stratified by age, sex, and weight category at inclusion, stratified by intervention and control groups.

	**Change in BMIz per year (95% CI)**	**Change in BMIz per year (95% CI)**
	**Intervention group**	**Control group**
Age		
≤11		
>11		
Sex		
Boys		
Girls		
Weight category		
Overweigh		
Obesity		

Abbreviations: BMIz, body mass index z-score

Supplementary material

REFERENCES

1. Collaboratio n NCDRF. Worldwide trends in body-mass index, underweight, overweight, and obesity from 1975 to 2016: a pooled analysis of 2416 population-based measurement studies in 128.9 million children, adolescents, and adults. Lancet. 2017;390(10113):2627-42.
2. Zhang X, Liu J, Ni Y, Yi C, Fang Y, Ning Q, et al. Global Prevalence of Overweight and Obesity in Children and Adolescents: A Systematic Review and Meta-Analysis. JAMA Pediatr. 2024;178(8):800-13.
3. Jebeile H, Kelly AS, O'Malley G, Baur LA. Obesity in children and adolescents: epidemiology, causes, assessment, and management. Lancet Diabetes Endocrinol. 2022;10(5):351-65.
4. Rasmussen M, Damsgaard MT, Morgen CS, Kierkegaard L, Toftager M, Rosenwein SV, et al. Trends in social inequality in overweight and obesity among adolescents in Denmark 1998-2018. Int J Public Health. 2020;65(5):607-16.
5. Lister NB, Baur LA, Felix JF, Hill AJ, Marcus C, Reinehr T, et al. Child and adolescent obesity. Nat Rev Dis Primers. 2023;9(1):24.
6. Tine M. Eriksen JG, Matvei Andersen, and Mette T. Jensen. The Social Cost of Childhood Overweight and Obesity – Results on Health Care
Usage, Well-Being, Education, and Labor Market Outcomes Until the Age of 21. VIVE. 2024.
7. Bjerregaard LG, Adelborg K, Baker JL. Change in body mass index from childhood onwards and risk of adult cardiovascular disease(). Trends Cardiovasc Med. 2020;30(1):39-45.
8. Bjerregaard LG, Jensen BW, Angquist L, Osler M, Sorensen TIA, Baker JL. Change in Overweight from Childhood to Early Adulthood and Risk of Type 2 Diabetes. N Engl J Med. 2018;378(14):1302-12.
9. Olsen NJ, Ostergaard JN, Bjerregaard LG, Hoy TV, Kierkegaard L, Michaelsen KF, et al. A literature review of evidence for primary prevention of overweight and obesity in healthy weight children and adolescents: A report produced by a working group of the Danish Council on Health and Disease Prevention. Obes Rev. 2023:e13641.
10. Ali A, Al-Ani O, Al-Ani F. Children's behaviour and childhood obesity. Pediatr Endocrinol Diabetes Metab. 2024;30(3):148-58.
11. Hollensted M, Fogh M, Schnurr TM, Kloppenborg JT, Have CT, Ruest Haarmark Nielsen T, et al. Genetic Susceptibility for Childhood BMI has no Impact on Weight Loss Following Lifestyle Intervention in Danish Children. Obesity (Silver Spring). 2018;26(12):1915-22.
12. Su X, Hassan MA, Kim H, Gao Z. Comparative effectiveness of lifestyle interventions on children's body composition management: A systematic review and network meta-analysis. J Sport Health Sci. 2024;14:101008.
13. Davies AL SF, Caldwell DM, et al. Factors associated with the effectiveness of interventions to prevent obesity in children: a synthesis of evidence from 204 randomised trials. BMJ Public Health. 2025.
14. Russell SJ, Mytton OT, Viner RM. Estimating the effects of preventive and weight-management interventions on the prevalence of childhood obesity in England: a modelling study. Lancet Public Health. 2023;8(11):e878-e88.
15. Kelleher E, Davoren MP, Harrington JM, Shiely F, Perry IJ, McHugh SM. Barriers and facilitators to initial and continued attendance at community-based lifestyle programmes among families of overweight and obese children: a systematic review. Obes Rev. 2017;18(2):183-94.
16. Epstein LH, Wilfley DE, Kilanowski C, Quattrin T, Cook SR, Eneli IU, et al. Family-Based Behavioral Treatment for Childhood Obesity Implemented in Pediatric Primary Care: A Randomized Clinical Trial. Jama. 2023;329(22):1947-56.
17. Jorgensen RM, Stovring H, Ostergaard JN, Hede S, Svendsen K, Vestergaard ET, et al. Long-Term Change in BMI for Children with Obesity Treated in Family-Centered Lifestyle Interventions. Obes Facts. 2024:1-12.
18. Margetin CA, Rigassio Radler D, Thompson K, Ziegler J, Dreker M, Byham-Gray L, et al. Anthropometric Outcomes of Children and Adolescents Using Telehealth with Weight Management Interventions Compared to Usual Care: A Systematic Review and Meta-analysis. J Am Nutr Assoc. 2022;41(2):207-29.
19. Whitehead L, Kabdebo I, Dunham M, Quinn R, Hummelshoj J, George C, et al. The effectiveness of nurse-led interventions to prevent childhood and adolescent overweight and obesity: A systematic review of randomised trials. J Adv Nurs. 2021;77(12):4612-31.
20. Tamayo MC, Dobbs PD, Pincu Y. Family-Centered Interventions for Treatment and Prevention of Childhood Obesity in Hispanic Families: A Systematic Review. J Community Health. 2021;46(3):635-43.
21. Hoare JK, Lister NB, Garnett SP, Baur LA, Jebeile H. Weight-neutral interventions in young people with high body mass index: A systematic review. Nutr Diet. 2023;80(1):8-20.
22. Tully L, Arthurs N, Wyse C, Browne S, Case L, McCrea L, et al. Guidelines for treating child and adolescent obesity: A systematic review. Front Nutr. 2022;9:902865.
23. Mead E, Brown T, Rees K, Azevedo LB, Whittaker V, Jones D, et al. Diet, physical activity and behavioural interventions for the treatment of overweight or obese children from the age of 6 to 11 years. Cochrane Database Syst Rev. 2017;6(6):CD012651.

24. Al-Khudairy L, Loveman E, Colquitt JL, Mead E, Johnson RE, Fraser H, et al. Diet, physical activity and behavioural interventions for the treatment of overweight or obese adolescents aged 12 to 17 years. Cochrane Database Syst Rev. 2017;6(6):CD012691.
25. Gow ML, Tee MSY, Garnett SP, Baur LA, Aldwell K, Thomas S, et al. Pediatric obesity treatment, self-esteem, and body image: A systematic review with meta-analysis. Pediatr Obes. 2020;15(3):e12600.
26. Molenberg FJM, Smit MS, Nieboer D, Voortman T, Jansen W. The long-term effects of a school-based intervention on preventing childhood overweight: Propensity score matching analysis within the Generation R Study cohort. Pediatr Obes. 2025;20(3):e13200.
27. Sundhedsstyrelsen. Vejledning om forebyggende sundhedsydelser til børn og unge. Sundhedsstyrelsen. 2025.
28. Eg M, Frederiksen K, Vamosi M, Lorentzen V. How family interactions about lifestyle changes affect adolescents' possibilities for maintaining weight loss after a weight-loss intervention: a longitudinal qualitative interview study. J Adv Nurs. 2017;73(8):1924-36.
29. Authority TDH. Anbefalinger Livsstilsintervention ved svær overvægt. 2024;ver-211019.Høringsudkast.
30. Cole TJ, Lobstein T. Extended international (IOTF) body mass index cut-offs for thinness, overweight and obesity. Pediatr Obes. 2012;7(4):284-94.
31. Bliddal M, Broe A, Pottegård A, Olsen J, Langhoff-Roos J. The Danish Medical Birth Register. Eur J Epidemiol. 2018;33(1):27-36.
32. Obesity: preventing and managing the global epidemic. Report of a WHO consultation. World Health Organ Tech Rep Ser. 2000;894:i-xii, 1-253.
33. Glasscock DJ, Andersen JH, Labriola M, Rasmussen K, Hansen CD. Can negative life events and coping style help explain socioeconomic differences in perceived stress among adolescents? A cross-sectional study based on the West Jutland cohort study. BMC Public Health. 2013;13:532.
34. Lindholdt L, Labriola M, Andersen JH, Kjeldsen MZ, Obel C, Lund T. Perceived stress among adolescents as a marker for future mental disorders: A prospective cohort study. Scand J Public Health. 2022;50(3):412-7.
35. Christiansen M, Hansen CD, Glasscock D, Andersen JH. [Social inequality and health in adolescents]. Ugeskr Laeger. 2010;172(11):857-63.
36. Poulsen PH, Biering K, Winding TN, Nohr EA, Petersen LV, Ulijaszek SJ, et al. How does psychosocial stress affect the relationship between socioeconomic disadvantage and overweight and obesity? Examining Hemmingsson's model with data from a Danish longitudinal study. BMC Public Health. 2019;19(1):1475.
37. Billeskov L, Labriola M, Stabell CL, Dieckmann MH, Jensen NH, Winding TN, et al. Coping strategies in adolescence and labour-market participation in young adulthood: A prospective birth cohort study. Scand J Public Health. 2020;48(5):537-43.
